# A non-invasive intratracheal inoculation method for the study of pulmonary melioidosis

**DOI:** 10.3389/fcimb.2012.00164

**Published:** 2012-12-20

**Authors:** David A. Revelli, Julie A. Boylan, Frank C. Gherardini

**Affiliations:** Laboratory of Zoonotic Pathogens, National Institute of Allergy and Infectious Diseases, Rocky Mountain LaboratoriesHamilton, MT, USA

**Keywords:** pulmonary melioidosis, melioidosis, non-invasive inoculation method, intratracheal inoculation, *Burkholderia pseudomallei*

## Abstract

Pulmonary melioidosis, a disease manifestation caused by the bacterium *Burkholderia pseudomallei*, has been studied using aerosols or intranasal (IN) inoculation in small animal models. Both have inherent disadvantages which may not accurately model primary pulmonary melioidosis in humans. Intratracheal inoculation (IT) by direct visualization of the tracheal opening offers an alternative technique for infection that overcomes the disadvantages of aerosol and IN challenge. In this study, we describe a method which requires relatively inexpensive equipment, little training, and is compliant with the operational constraints of a BSL3 laboratory. Results obtained using trypan blue demonstrated that an inoculum can be accurately delivered into the lungs of mice within a biosafety cabinet (BSC). Whole body imaging and histopathology confirmed that mice inoculated intratracheally with *B. pseudomallei* develop the primary focus of infection in the lungs, and not the nasal passages which can lead to invasion of the central nervous system and potential neurologic complications. Further, based on colony counts and bioluminescent imaging, dissemination to secondary organs occurred as expected. Taken together, this intratracheal method of inoculation fulfills four goals: (1) to accurately deliver *B. pseudomallei* into the lungs of the animal model, (2) to avoid potentially confounding complications due to primary infections at sites other than the lung, (3) to maintain normal organ dissemination, and (4) to be BSL3 compliant.

## Introduction

The bacterium *Burkholderia pseudomallei* is a gram-negative bacterial pathogen that is the etiologic agent of the disease melioidosis. Melioidosis is a serious and often fatal disease of humans and animals which is endemic in Southeast Asia, northern Australia, and other tropical areas of the world (Cheng and Currie, 2005). Infection usually follows percutaneous inoculation or inhalation of the bacterium that are present in soil and surface water. The disease may be localized or disseminated, and virtually any organ system can be affected, resulting in a wide range of symptoms that are often mistaken for other illnesses, such as tuberculosis or pneumonia (Loveleena et al., 2004). The overall mortality rate of melioidosis in endemic areas may reach as high as 50% (White, 2003).

While community-acquired cases of melioidosis are most likely a consequence of the bacterium in soil or water entering the host through cuts or skin abrasions, the bacterium is much more infective via the inhalation route as determined in small animal models of disease (Titball et al., 2008). This is possibly consistent with the higher incidence of melioidosis during monsoon season, when surface waters are aerosolized by severe weather, and the higher incidence of reported cases in healthy American helicopter crews during the Vietnam War, as a consequence of the inhalation of dusts containing bacteria (Howe et al., 1971; Cheng and Currie, 2005). The potential for the bacterium to cause disease after inhalation, in addition to the high mortality associated with the disease and the intrinsic antibiotic resistance, has resulted in the inclusion of this pathogen on the list of potential biological warfare and bioterrorism agents, and the classification of *B. pseudomallei* as a Tier 1 Select Agent by the Centers for Disease Control (CDC) (Rotz et al., 2002). Since the anthrax attacks of 2001, the inhalation route has been given special interest because it is a likely route to be exploited by bioterrorists.

Pulmonary melioidosis, caused by either inhalation or hematogenesis spread, is an important sequela of the disease and is present in over 50% of diagnosed cases (Cheng and Currie, 2005; Currie et al., 2010). It presents as a febrile condition which includes coughing, respiratory distress, and production of sputum (Vietri and Deshazer, 2007). Pulmonary melioidosis, caused by inhalation, is a particularly pertinent area of study in the disease process as the lungs represent the potential point of inoculation and dissemination into the body during a bioterrorist attack.

The most commonly used animal models in the study of melioidosis are the hamster and the mouse (Warawa, 2010). The mouse is the most attractive model given its ease of use and the tools available for studying genetics and physiology Both the acute and the chronic forms of the disease can be studied in mice using different genetic backgrounds, BALB/c and C57BL/6, respectively (Leakey et al., 1998). Generally, pulmonary melioidosis has been studied using either aerosolization or intranasal (IN) inoculation as the route of infection (Owen et al., 2009; Warawa, 2010; Thomas et al., 2012). Both methods have advantages and disadvantages for animal research. The aerosolization method most closely resembles the route of natural infection in humans, but depending on particle size and the anatomical parameters of the animal, the procedure requires a higher titer of bacteria to achieve study dosages within the lungs (Brain et al., 1976; Munder et al., 2002). In the case of IN inoculation, the inoculum is applied to the nares of the mouse and is respired through the nasal passage into the lungs, but the majority of the inoculum remains within the nasal passages or is removed through the gastrointestinal tract; therefore, only a small portion of the inoculum is translocated into the lungs (Watson et al., 1969; Su et al., 2004). In addition, these routes of infection often result in high rates of infections of the nasal-associated lymphoid tissue (NALT) and olfactory tissues (CNS) in mice where such infections are uncommon in humans (Currie et al., 2000; Owen et al., 2009; Warawa et al., 2011). Consequently, these two methods may not accurately model inhalational melioidosis in humans.

Intratracheal inoculation (IT) offers another potential route of infection to study pulmonary melioidosis that avoids the potential CNS infection associated with aerosolization and IN inoculation by placing the inoculum directly into the trachea, thereby bypassing the mouse nasal passages. IT inoculation has the advantages of being able to introduce a known quantity of sample directly into the lungs of the subject unlike either IN inoculation or aerosolization (Watson et al., 1969; Driscoll et al., 2000). Both surgical and non-invasive methods have been used to introduce various substances and bacteria directly into the lungs of mice to study toxicity or virulence (Ho and Furst, 1973; Brain et al., 1976; Lawrence et al., 1977; Starcher and Williams, 1989; Hastings and Summers-Torres, 1999; Driscoll et al., 2000; Munder et al., 2002; Su et al., 2004; Guilbault et al., 2005; Lacher et al., 2010). Non-invasive methods of IT inoculation present less risk to the investigator, require less training, require less time for the procedure, and have less specialized equipment than aerosol delivery systems. The above points are especially important for studies with *B. pseudomallei* which necessitate the use of a biosafety cabinet (BSC) within a biosafety level 3 (BSL3) laboratory.

The purpose of this study was to develop a non-invasive intratracheal delivery method that fulfilled four main goals: (1) accurate delivery of the inoculum into the lungs of the animal, (2) models the pulmonary route of infection avoiding potential confounding complications due to neurologic melioidosis, (3) maintain organ dissemination as seen during the typical *B. pseudomallei* infection, and (4) is BSL3 compliant.

## Materials and methods

### Bacterial strains and growth

*B. pseudomallei* DD503 containing the *luxCDABE* (JW280) operon from *Photorhabdus luminescens* (Warawa et al., 2011), was grown in Luria broth-Lennox (Difco) supplemented with 4% glycerol (LB4G) at 37°C with shaking for all experiments. Brucella agar (Difco), supplemented with 4% glycerol (B4G), was used to recover bacteria from organs. Modified Ashdown's agar (Ashdown, 1979) containing polymyxin B (100 μg/ml) was used to recover bacteria from the lungs. All experiments conducted with *B. pseudomallei* were performed in a BSL3 laboratory using Select Agent compliant BSL-3/ABSL-3 containment and procedures.

### Experimental animals

For trypan blue staining of the lungs, mice bred at Rocky Mountain Laboratories, NIAID/NIH, designated RML were used. For infections and imaging using *B. pseudomallei*, female, BALB/c, 8- to 10-week-old mice were obtained from Charles River Laboratories (Wilmington, MA). In preparation for imaging and before inoculation, mice were anesthetized using 3–4% isoflurane, and the fur removed on their dorsal thoracic cavities by shaving to reduce interference during imaging. All studies were performed in accordance with the Institutional Animal Care and Use Committee's guidelines at Rocky Mountain Laboratories, NIH, Hamilton, MT.

### Equipment

#### Backboard

**Figure 1:**
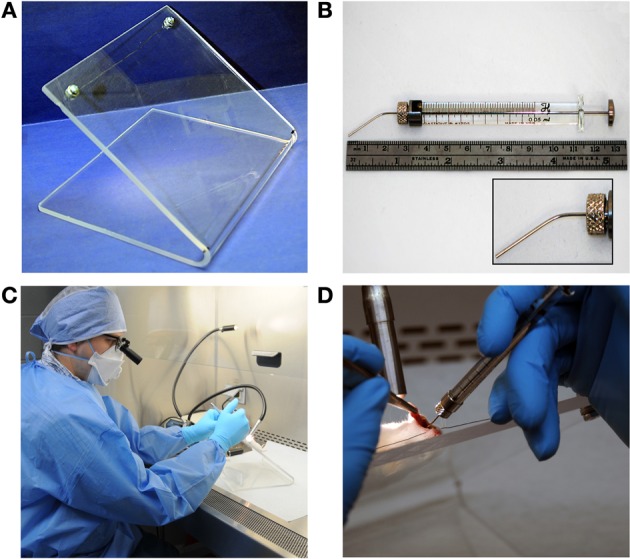
**Equipment. (A)** Backboard constructed of plexiglass with wire strung between screws. **(B)** Gastight syringe (50 μl total volume) with bent and blunted 22s gauge needle. **(C)** General equipment arrangement within a biosafety cabinet. **(D)** Instillation procedure with lightsource placement, withdrawn tongue, and placement of the gastight syringe.

Plexiglass (6 mm thick) was cut to measure approximately 42 cm in length by approximately 22 cm in width and bent to a 45° acute angle. Two holes were drilled on each side of the backboard 2 cm from the top and side edge. Galvanized metal-style screws, nuts, and washers were used to secure a stainless steel wire strung from one screw to the other from which a mouse could be hung by its incisors (Figure 1A). For surface decontamination, 2% Roccal-D (Pfizer Inc, New York) was regularly used as 70% ethanol caused cracking and eventual breakage of the backboard.

#### Gastight syringe and needle

Fifty microliter gastight syringes with removable needles (model 1705 RN SYR) were obtained from the Hamilton Company (Reno, NV). Gastight syringes were used in conjunction with custom 22s gauge, small hub, blunt, and needles approximately 25 mm in length. Needles were bent in order to facilitate direct instillation of bacteria into the trachea (Figure 1B).

#### Surgical loupes

Dental/medical loupes (6X, 550 mm working distance) were used to directly visualize the larynx and tracheal opening through the glass sash of the BSC during instillation.

### Intratracheal inoculation

**Figure 2:**
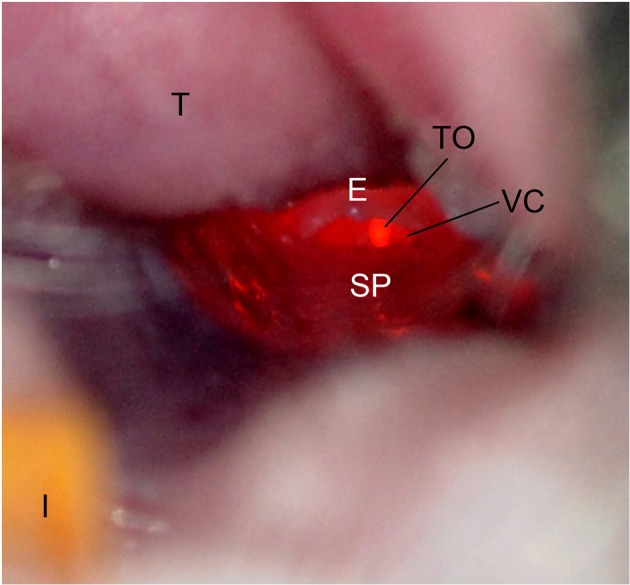
**Mouse oropharynx anatomy.** The mouse in the supine position with the incisors (I) at the bottom and the tongue (T) located at the top of the figure. The tracheal opening (TO), epiglottis (E), vocal chords (VC), and soft palate (SP) are labeled.

Mice were anesthetized using an isoflurane vaporizer and induction chamber (Matrx VIP3000, Midmark, Orchard Park, NY). After animals became recumbent, mice were injected intraperitoneally (IP) with 80 μl of a cocktail of ketamine (25 mg/ml) and xylazine (1.2 mg/ml) and returned to the isoflurane induction chamber. For inoculation, animals were removed from the chamber and suspended on the plexiglass backboard in the supine position by their incisors (Figure 1D). A lightsource (Dolan-Jenner Model MI-150 Illuminator, VWR International, Bridgeport, NJ) was used to illuminate the tracheal opening by positioning a fiber optic arm over the front side of the throat (Figure 1D). The mouth was opened and forceps were used to extend the tongue out of the mouth and to the side and the needle was used to gently reposition the soft palate to expose the tracheal opening (Figure 1D). The needle was inserted between the vocal cords (Figure 2) to inoculate.

To determine lobular involvement, intrapulmonary staining was achieved by the introduction of 20 μl of 0.4% trypan blue (Gibco) using the procedure described above. RML mice were euthanized and lungs excised. The effectiveness of the technique was determined empirically by observing the number of lobes exhibiting trypan blue staining.

### Bacterial challenge and bioluminescent imaging

Bacteria were inoculated from overnight cultures into fresh LB4G and incubated at 37°C with shaking to an OD_600_ of 0.4. Animals (*n* = 3 − 4) were inoculated with 1 × 10^4^ CFUs of *B. pseudomallei* JW280 in a total volume of 20 μl for IT inoculation, or 30 μl in the left nare for IN inoculation. At time 0, 24, 48, and 72 h post-infection, mice were imaged with an IVIS Lumina imaging system (Caliper Lifesciences, Hopkinton, MA) using a Xenogen animal isolation chamber (Caliper Lifesciences, Hopkinton, MA) for biocontainment during the imaging procedure.

For imaging, mice were placed inside the animal isolation chamber within a BSL3-compliant BSC. The isolation chamber was surface decontaminated, removed to the IVIS imaging chamber and connected to the in-chamber anesthesia delivery system (Xenogen XGI-8 Gas Anesthesia System, Caliper Lifesciences, Hopkinton, MA). Isoflurane was applied to the chamber until the mice became recumbent. The isolation chamber was then placed into the imaging chamber and reconnected to the anesthesia delivery system. Bioluminescence was measured using 5 min exposures.

All mice were euthanized and dissected immediately at 72 h post-infection. *Ex vivo* images of the lungs, liver, and spleen were obtained by containing organs in a petri dish in the isolation chamber. The isolation chamber was positioned in the imaging cabinet and organs were imaged using 5 min exposures.

Bioluminescence was quantitated using total radiance of the dorsal thoracic and nasal regions of infected animals using Living Image software 3.0 (Caliper Lifesciences). The units of average radiance are photons/second/square centimeter/solid angle (p/s/cm2/sr). Graphing and statistical analysis was performed using an unpaired *t*-test analysis in GraphPad Prism 5 (GraphPad Software Inc.).

### Bacterial enumeration

Organs were homogenized using a Mini-Beadbeater-1 (BioSpec Products, Inc., Bartlesville, OK) and Lyzing Matrix H (MP Biomedicals, Solon, OH). Lyzing Matrix H tubes containing 2 mm glass and 2 mm yellow zirconium oxide beads were used to homogenize tissue samples. The tubes were filled with 0.5 ml of 1X PBS (Gibco) and preweighed before entering the BSL3. After dissection, organs were placed into tubes and weighed. Organs were homogenized on high setting for 10 s serial diluted, and plated onto B4G or Modified Ashdown's Media (lungs). All steps involving the bacterium were performed in a BSC.

### Histologic analysis

Animals were euthanized and tissues were harvested for histopathologic analysis. At dissection, lungs were inflated with 10% formalin and then immersed in formalin for a minimum of 48 h. Tissues were fixed in formalin, processed, and stained with hematoxylin and eosin. Mouse heads, as part of processing, were decalcified using EDTA in 20% sucrose and sectioned. Histopathology of the nasal nares, brain, lung, liver, and spleen were scored as previously described (Warawa et al., 2009). Statistical analysis was performed using GraphPad Prism 5 (GraphPad Software Inc.).

## Results

### Targeting the lung

As a first step to evaluating the use of IT inoculation for mouse infections, we wanted to ensure the accurate targeting of the lung within the confines of BSL3 operating procedures. To do this, 10 mice were inoculated with trypan blue stain. In order to achieve inoculation directly into the lungs, dental/surgical loupes (working distance 55 cm) are used to visualize and differentiate the anatomy of the larynx of the mice. The depth of field (ca. 10 mm) of the dental/surgical loupes allows the visualization of the mouse larynx and insertion of the needle into the tracheal opening. As Figure 2 demonstrates, when the lightsource is positioned over the front of the throat, the tracheal opening and the vocal chords are easily differentiated from other anatomical structures. The soft palate was repositioned using the syringe by gently depressing downward toward the dorsal side of the mouse, exposing the tracheal opening.

**Figure 3:**
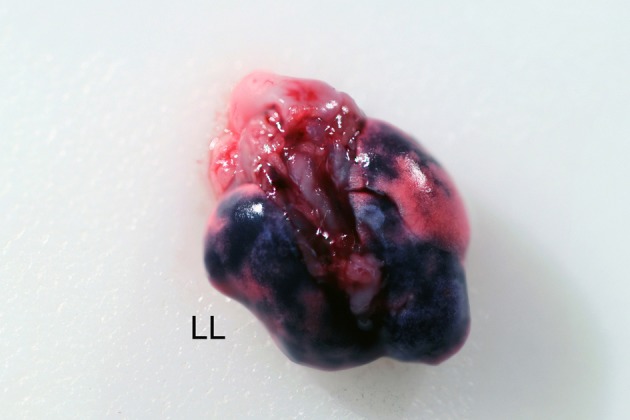
**Intratracheal delivery of trypan blue.** Mice were anesthetized with 2.5% isoflurane and instilled with 20 μl of a 0.4% trypan blue solution. Mice were euthanized and the lungs harvested. Left lobe (LL) is labeled for orientation.

It should be noted that different anesthetics alter the configuration of murine oropharyngeal anatomy. The tracheal opening remained occluded and difficult to identify when the ketamine/xylazine cocktail was used as the sole anesthetic. When isoflurane was used as the sole anesthetic, the tracheal opening remained unobscured and easily identifiable. However, use of isoflurane as the sole anesthetic was judged to be problematic due to the requirement of a continuous delivery because of the short-lived effects of the anesthesia. This could allow the animal to become active during inoculation and the use of a continuous delivery system could obstruct the view of the oropharynx. To mitigate unnecessary risk and abolish maintenance of the swallowing reflex, the ketamine/xylazine cocktail was used in addition to isoflurane. Animals were injected with the ketamine/xylazine cocktail, allowed to become recumbent, and immediately prior to performing the procedure in the BSC were induced with 2.5% isoflurane to abolish the swallowing reflex. The result of the trypan blue stain inoculation is shown in Figure 3. Trypan blue was observed in the lungs of all 10 mice. Importantly, inoculation by this method will allow for the delivery of an agent into multiple lobes of the lungs (Figure 3).

### Bioluminescent imaging of intratracheal and intranasal inoculations

**Figure 4:**
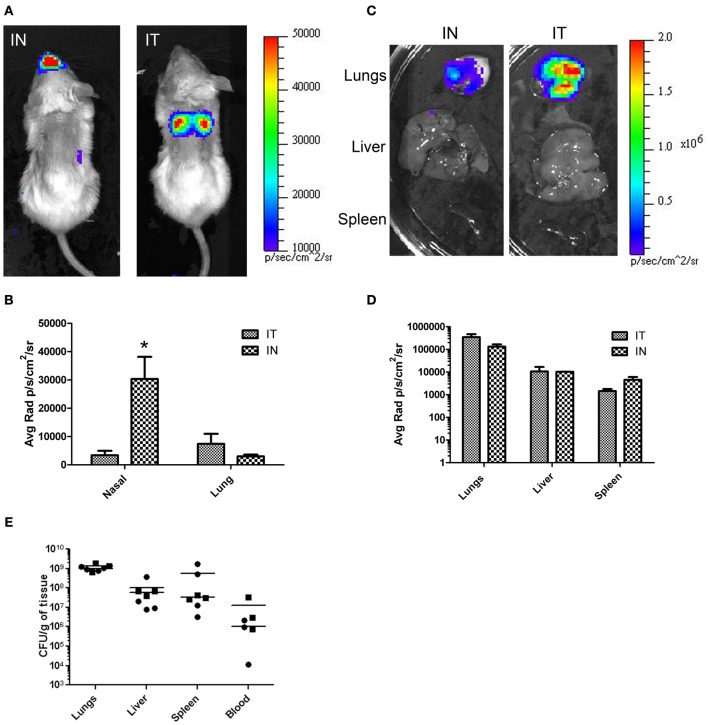
***In vivo* and *ex vivo* imaging of *B. pseudomallei* infections comparing IT and IN inoculation methods.** Mice were infected with 10^4^ cfu of *B. pseudomallei* JW280. **(A)** Mice were inoculated by either IT or IN inoculation methods and imaged using an IVIS Lumina system at 72 h. **(C)** At 72 h, mice were euthanized and organs were harvested and imaged. (**B** and **D**) Light emission using average radiance (photons/second/square centimeter/steradian) from **(B)** whole-body and **(D)** harvested organs as determined using Living Image 3.0 software. All images were normalized to the same average radiance scale. **(E)** Bacterial burden determination in homogenized organs. ^*^p = 0.0279.

The common practice of mouse IN inoculations typically results in infections of the nasal cavity and invasion of the CNS not normally seen in humans. To demonstrate the effectiveness of IT inoculations to specifically target the lungs without CNS involvement, mice were challenged with a bioluminescent strain of *B. pseudomallei*, JW280, and imaged over 3 days. For comparison, the mice were also inoculated using IN inoculation method. The relative bioluminescent intensity is indicative of the bacterial cell density, therefore, the relative light intensity can be used as a measure of bacterial infection. At times 0, 24, and 48 h, no significant luminescence was observed in mice from either challenge (data not shown). At 72 h, significant luminescence was observed in both IT and IN inoculated mice. In IT inoculated mice, bioluminescence was observed emanating mainly from the thoracic region of the mouse with little or no luminescence observed from the nasal cavity region (Figure 4A, panel IT). In contrast, mice inoculated by the IN inoculation method showed significant amounts of luminescence in the nasal region and relatively little luminescence from the thoracic region (Figure 4A, panel IN). The difference in luminescence between the two procedures was quantified by calculating the average radiance for the thoracic and nasal regions of each mouse. The results are shown in Figure 4B. In mice infected by IT inoculation, the thoracic regions exhibited no significant increase in average radiance (11,373 p/s/cm^2^/sr) compared to the average radiance in mice infected by IN inoculation (6970 p/s/cm^2^/sr; *p* = 0.2849), indicating that IT inoculation results in infectivity of the lung at the same level as the commonly used IN inoculation procedure. Importantly, in mice infected by IN inoculation, the nasal regions exhibited approximately 5-fold higher average radiance (33,767 p/s/cm^2^/sr) compared to the average radiance in mice infected by IT inoculation (6,760 p/s/cm^2^/sr) demonstrating a marked decrease in nasal infections when the mice are inoculated IT.

**Figure 5:**
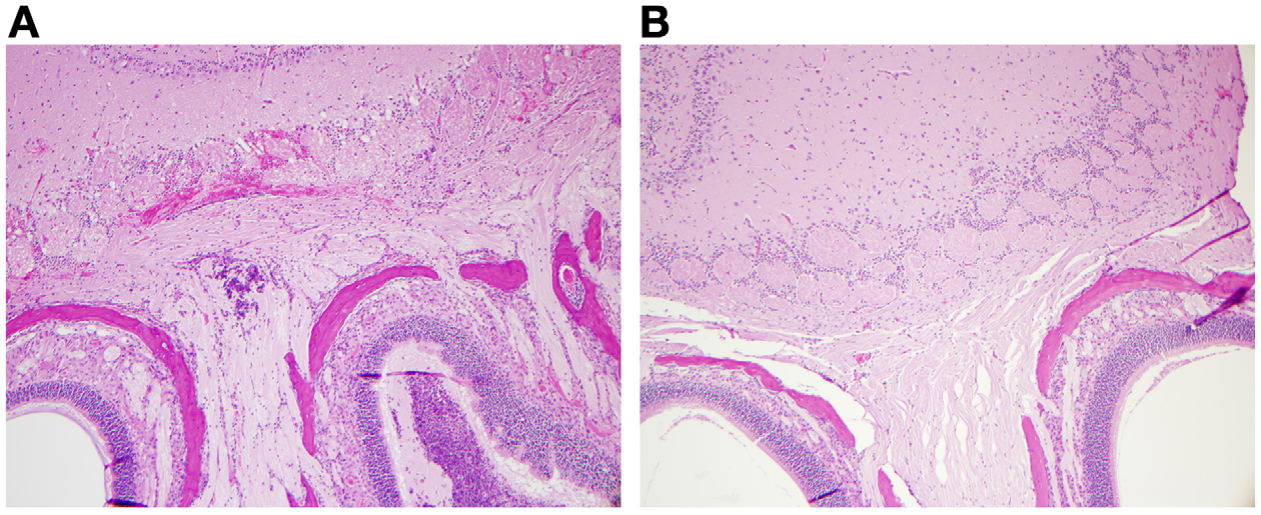
**Histopathology of IN infected mice demonstrated damage of the CNS and nasal epithelium.** Nasal and olfactory tissue from mice infected by IT or IN inoculation with 10^4^ cfu of *B. pseudomallei* JW280. The heads of **(A)** IN-infected or **(B)** IT-infected mice were harvested at 72 h, fixed with formalin, embedded, and stained with hematoxylin and eosin. Histological sections were imaged by bright field microscopy.

To further characterize the nasal infections and determine CNS infiltration, histopathology of the nasal cavity and the brain was assessed using tissues collected 72 h post-inoculation. The results are shown in Figure 5. In mice infected by IN inoculation, inflammatory cell infiltrates and hemorrhaging are present in the olfactory bulbs (CNS) and in the olfactory nerve filaments descending from the CNS through the cribriform plate into the nasal epithelium. Additionally, necrotic lesions present throughout the nasal epithelium (necrotizing rhinitis) and inflammatory exudate in sinus cavities indicate significant nasal infection and CNS infiltration (Figure 5A). In contrast, olfactory bulbs from IT infected mice exhibited no inflammation or hemorrhaging, normal nasal epithelium and no evidence of inflammatory exudate (Figure 5B).

The difference in luminescence was also determined in the organs of animals infected IT vs. IN inoculation. Mice were euthanized after whole-body imaging at 72 h, and the lungs, liver, and spleen were immediately harvested and imaged. The results are shown in Figure 4C. Luminescence was quantitated using the Living Image software (Caliper Lifesciences). In each case there was no significant difference in luminescence of the organs between inoculation by IT and IN (Figure 4D). To correlate light intensity to bacterial burden in the organs, the organs were homogenized, serial diluted, plated, and colonies counted. No significant difference in bacterial burden between methods (Figure 4E) was found for the lungs (unpaired *t*-test, *p* = 0.3767), the liver (unpaired *t*-test, *p* = 0.6906), the spleen (unpaired *t*-test, *p* = 0.3173), and the blood (unpaired *t*-test, *p* = 0.3405). Taken together, these results demonstrate that this method directly and accurately targets the lungs and results in a pulmonary infection at the same or higher levels than that of the more commonly used IN inoculation procedure. Furthermore, inoculation by IT does not result in primary CNS infection.

## Conclusions

The potential for outbreaks of melioidosis following a bioterrorist attack has led to increased interest in understanding associated virulence factors and disease course produced by the bacterium *B. pseudomallei*. Studies have been performed using several methods of inoculation to mimic different routes of infection such as subcutaneous and intraperitoneal (percutaneous), oral (ingestion), and IN and aerosol (inhalation) (Jeddeloh et al., 2003; Barnes and Ketheesan, 2005; Tan et al., 2008; Lever et al., 2009; Owen et al., 2009). Among these, inhalation is the most probable route for bioterrorist delivery of *B. pseudomallei*, which most likely would result in infections of the lung, i.e., pulmonary melioidosis. The most common inoculation methods utilized to mimic the airborne route are IN and aerosol. While both methods result in infections of the lung and exhibit normal organ dissemination, neurological abnormalities appear to be overrepresented, possibly due to the anatomical differences of the upper respiratory tract (URT) between mice and humans.

Rodents, unlike humans, are obligatory nose-breathers, and divergent evolution has equipped the animal with a URT developed for enhanced olfactory senses (Reznik, 1990). Thus, rodents have a greater relative surface area in the URT than humans possess (Warawa, 2010). Along with the greater surface area and enhanced olfaction, specific tissue types are likely to be overrepresented in the mouse compared to the human, such as olfactory-associated epithelium. The combination of a larger relative surface area with comparatively overrepresented tissues in the URT of rodents may complicate the study of *B. pseudomallei* infections by overrepresenting specific portals of entry depending on the route of inoculation. Any route of inoculation that depends on the normal inspiratory route of the rodent, such as IN or aerosol, could potentially favor these alternative portals of entry to the body. In addition, IN inoculation results in only a fraction of the bacteria reaching the lungs leaving a major proportion to localize in the URT (Munder et al., 2002; Su et al., 2004). Olfactory epithelium and the trigeminal nerve may serve as portals of entry to the CNS and, as such, IN inoculation has been utilized to study *B. pseudomallei* CNS infection (Owen et al., 2009). Neurologic abnormalities present during infection with *B. pseudomallei*, with or without evidence of direct invasion of the CNS, has been termed neurologic melioidosis and is considered “unusual” in humans, occurring in approximately 3–4% of all melioidosis cases (Currie et al., 2000, 2010; Koszyca et al., 2004; Owen et al., 2009). Because of this low incidence level, an animal model which frequently results in neurologic melioidosis may not accurately model inhalation melioidosis in humans. In fact, in humans, pulmonary melioidosis is the more common manifestation of disease resulting from inhalation of bacteria, with around half of all melioidosis patients presenting with pneumonia (Currie, 2003). Thus, a more accurate animal model would be one that specifically targets the lower respiratory tract resulting in pulmonary melioidosis in the mouse.

Intratracheal instillations have been used to deliver test materials directly into the lungs of mice in numerous studies (Ho and Furst, 1973; Brain et al., 1976; Lawrence et al., 1977; Lacher et al., 2010), but is not widely used to model mouse pulmonary melioidosis. Many of these reports detail methods of both invasive and non-invasive procedures of intratracheal instillation. However, to our knowledge there is no report in the literature of a method specifically suited for BSL3 compliance. The method detailed in this report fulfills four important goals of this study which are: (1) accurate delivery of the inoculum into the lungs of the animal, (2) models pulmonary route of infection avoiding potential confounding complications due to neurologic melioidosis, (3) maintains organ dissemination as seen during typical *B. pseudomallei* infection, and (4) is BSL3 compliant.

Since our lab primarily focuses on pulmonary melioidosis, IT inoculation was chosen as the method of infection in order to deliver a known number of bacteria directly to the lungs and thereby bypass the URT of the mouse. In initial experiments, the inoculations were performed as previously reported using the “blind” or “feel” approach. In this technique the inoculum is introduced by following the midline of the tongue to the back of the pharynx over the epiglottis and into the tracheal opening, not through visualization of the tracheal opening. Using this technique, we observed a low accuracy of instillation into the lungs of between 10 and 20%. The inoculum was generally found in the esophagus or in the extratracheal tissue due to perforation of the trachea. Based on these results, we determined that a direct visualization approach to introduce a sample would be more advantageous.

Most methods for non-invasive intratracheal inoculation require some basic equipment: (1) an apparatus to magnify the larynx for visualization of the tracheal opening, (2) a lightsource to illuminate the oropharynx, and (3) a device to deliver the inoculum. In addition, working with *B. pseudomallei* requires the user to perform all tasks within the confines of the BSC within a BSL3 laboratory. To best meet these requirements, our procedure utilizes an angled plexiglass backboard optimized for use in a BSC, dental/surgical loupes, a small-volume syringe with a modified needle, and a lightsource. In our experience, dental loupes are the best option to visualize the anatomy and precisely target the lungs because focus may be adjusted simply by head movement freeing the operator's hands from extraneous movements that might disrupt airflow in the BSC (Figure 1C). In addition, because the operator can see the needle entering the trachea, the actual inoculation procedure can be performed in under a minute. Using this method multiple lobes of the lung are involved (Figure 3). Although this may not exactly replicate inhalation, IT inoculation has been shown to reach deeper lung tissue than aerosol challenge and to deliver a known quantity of bacteria (Watson et al., 1969; Driscoll et al., 2000; Munder et al., 2002). It should be noted that this method assumes the use of an N95 or similar respirator and is not amenable for use with a PAPR.

Based on whole-body luminescent imaging, inoculation by IN and IT differed in the pattern of infection with respect to where the focus of infection was observed. Mice infected by IN inoculation appeared to have a primary focus of infection in the region of the nasal cavity at 72 h post-infection whereas the primary focus of infection at the same time for mice infected by IT inoculation was in the area of the thoracic cavity (Figure 4). This characteristic differential focus of infection can only be attributable to the method of inoculation since the same inoculum was given to both groups, and is supported by other studies which demonstrate a focus of infection in the nasal passages after IN inoculation (Owen et al., 2009; Massey et al., 2011; Warawa et al., 2011). Using histopathology, we observed significant hemorrhaging and necrosis at 72 h post-infection by IN inoculation with *B. pseudomallei* in the nasal epithelium and the olfactory bulbs, confirming the results of previous studies (Figure 5). This also indicated that inoculation by the IN route may overrepresent neurologic melioidosis in murine animal models, an uncommon presentation in humans. IT inoculation offers an alternative method of inoculation which bypasses nasal-associated routes of entry and focuses on the early infection in the lungs. In contrast to mice infected by IN inoculation, histopathology of the nasal passages and CNS of IT infected mice suggest the lack of involvement of those tissues during challenge with *B. pseudomallei* during early infection, since no inflammation or hemorrhaging was observed after IT inoculation (Figure 5). This offers the investigator the advantage of studying pulmonary melioidosis without potentially confounding neurological involvement, such as spread of bacteria through the NALT or CNS to other sites in the body.

There does appear to be a disparity between the amount of observed luminescence and bacterial burden of the lungs. We attribute this to differential deposition in the lungs due to the difference in methods. It has been demonstrated that infection by IT inoculation results in a localization of the inoculum deeper into the lung which resulted in bacteria being deposited in the portion of the lung closest to the skin (Watson et al., 1969). In mice inoculated by IN, the bacteria are deposited more shallowly (Watson et al., 1969). The depth and density of the tissue to be imaged within the body presents a major barrier to the detection of emitted light. Light emitted from deeper tissues is hard to detect because of the intervening tissues blocking the light. The difference in deposition of the bacteria between the methods would account for the difference in the observed luminescence.

To accurately study the progression of disease, it is important that the route of infection allows for the dissemination of the bacteria to secondary organs. Although the lungs may be the primary focus of infection, virtually any organ system can be affected, including the liver and spleen (White, 2003; Currie et al., 2010). Previous studies have demonstrated “normal” bacterial dissemination when animals were infected by the IN inoculation method, indicating that the lungs, liver, and spleen of animals were infected (Warawa et al., 2011). In this paper, we demonstrated that there is no significant difference between the IN and IT inoculation methods for bacterial burden in the lungs, liver, spleen, or blood. This suggested that dissemination using either method resulted in equivalent spread to those organs after 72 h (Figure 4). Taken together, these results indicated that the IT inoculation method of infection was a powerful tool in the study of pulmonary melioidosis. In addition, because this procedure requires minimal training and readily-available, inexpensive equipment, this method should be easily adaptable to the study of pulmonary infections for other select agents.

### Conflict of interest statement

The authors declare that the research was conducted in the absence of any commercial or financial relationships that could be construed as a potential conflict of interest.
